# Human platelet lysate in mesenchymal stromal cell expansion according to a GMP grade protocol: a cell factory experience

**DOI:** 10.1186/s13287-018-0863-8

**Published:** 2018-05-02

**Authors:** Valentina Becherucci, Luisa Piccini, Serena Casamassima, Silvia Bisin, Valentina Gori, Francesca Gentile, Riccardo Ceccantini, Elena De Rienzo, Barbara Bindi, Paola Pavan, Vanessa Cunial, Elisa Allegro, Stefano Ermini, Francesca Brugnolo, Giuseppe Astori, Franco Bambi

**Affiliations:** 1 Cell Factory Meyer, “A. Meyer” University Children’s Hospital, Florence, Italy; 2 Transfusion Medicine and Cell Therapy Unit, “A. Meyer” University Children’s Hospital, Florence, Italy; 3 Advanced Cellular Therapy Laboratory, Department of Cellular Therapy and Hematology, San Bortolo Hospital, Vicenza, Italy

**Keywords:** Cell factory, Good manufacturing practice, Mesenchymal stromal/stem cells, Platelet lysate, Fetal bovine serum, Advanced therapy medicinal products

## Abstract

**Background:**

The use of platelet lysate (PL) for the ex-vivo expansion of mesenchymal stromal/stem cells (MSCs) was initially proposed by Doucet et al. in 2005, as an alternative to animal serum. Moreover, regulatory authorities discourage the use of fetal bovine serum (FBS) or other animal derivatives, to avoid risk of zoonoses and xenogeneic immune reactions. Even if many studies investigated PL composition, there still are some open issues related to its use in ex-vivo MSC expansion, especially according to good manufacturing practice (GMP) grade protocols.

**Methods:**

As an authorized cell factory, we report our experience using standardized PL produced by Azienda Ospedaliero Universitaria Meyer Transfusion Service for MSC expansion according to a GMP grade clinical protocol. As suggested by other authors, we performed an in-vitro test on MSCs versus MSCs cultured with FBS that still represents the best way to test PL batches. We compared 12 MSC batches cultured with DMEM 5% PL with similar batches cultured with DMEM 10% FBS, focusing on the MSC proliferation rate, MSC surface marker expression, MSC immunomodulatory and differentiation potential, and finally MSC relative telomere length.

**Results:**

Results confirmed the literature data as PL increases cell proliferation without affecting the MSC immunophenotype, immunomodulatory potential, differentiation potential and relative telomere length.

**Conclusions:**

PL can be considered a safe alternative to FBS for ex-vivo expansion of MSC according to a GMP grade protocol. Our experience confirms the literature data: a large number of MSCs for clinical applications can be obtained by expansion with PL, without affecting the MSC main features. Our experience underlines the benefits of a close collaboration between the PL producers (transfusion service) and the end users (cell factory) in a synergy of skills and experiences that can lead to standardized PL production.

**Electronic supplementary material:**

The online version of this article (10.1186/s13287-018-0863-8) contains supplementary material, which is available to authorized users.

## Background

Mesenchymal stromal/stem cells (MSCs) represent one of the most promising tools in cellular therapy and regenerative medicine due to their immunomodulatory and regenerative properties [1–7]. Another emerging clinical application of mesenchymal stromal cells is represented by the targeted delivery of chemotherapeutic agents to neoplastic cells, maximizing the cytotoxic activity against cancer cells and minimizing collateral damage to non-neoplastic tissues [8, 9].

MSCs can be easily isolated from bone marrow (BM) thanks to their capacity to adhere and proliferate on plastic supports. The main characteristics of MSCs were described by the International Society for Stem Cell Therapy (ISCT) in 2006 [10]: they must be plastic-adherent when maintained in standard culture conditions, they must express CD105, CD73 and CD90 and must be negative for hematopoietic markers such as CD45, CD34, CD14 and HLA-DR, and finally they must be able to differentiate via osteoblastic, adipocytic and chondrocytic pathways in vitro.

In the last decade, the increasing use of MSCs as advanced therapy medicinal products (ATMP) has led to production processes that need to meet good manufacturing practice (GMP) [11] in order to ensure product safety and efficacy. Moreover, MSCs only represent 0.01–0.001% of total bone marrow mononucleated cells (BMNCs) [12] and ex-vivo expansion is necessary to obtain at least 0.5 × 10^6^–5 × 10^6^ MSCs/kg in clinical trials [13–15].

Basal growth media used for MSC expansion are supplemented with multiple nutrients and growth factors, and fetal bovine serum (FBS) has long been the gold standard medium supplement for laboratory-scale MSC culture. However, FBS has a poorly characterized composition, it can be a source of xenogenic antigens and zoonotic infections, and the lack of standardization of FBS preparations may affect cell culture performance [16].

For all of these reasons, the regulatory authorities discourage the use of FBS or other animal derivatives for cell expansion [17, 18], especially for clinical GMP production. In order to find a valid alternative to FBS for clinical MSC expansion, several studies have evidenced the possibility of replacing FBS with platelet lysate (PL) as it contains a plethora of growth-promoting factors [19, 20]. Although the use of human-derived blood materials alleviates the immunologic risk of FBS, the possibility of transmitting blood-borne viruses remains, especially when materials from multiple donors are pooled to minimize biological variability and to obtain a sufficient volume for therapeutic-scale MSC expansion.

### Considerations for use of PL in clinical MSC expansion

The use of PL for the ex-vivo MSC expansion was initially proposed by Doucet et al. [21] in 2005 as a source of bioactive molecules and growth factors released from α-granules after platelet rupture. Several studies [21, 22] investigated PL composition, finding inside a large number of growth factors where the most significant are certainly platelet-derived growth factor (PDGF), epidermal growth factor (EGF), insulin-like growth factor (IGF), transforming growth factor (TGF) and fibroblast growth factor 2 (FGF 2), all factors with strong mitogenic action. Platelet lysate also contains cytokines and chemokines like interleukin (IL)-1β, IL-2, IL-6, IL-10, IL-12p70, 1 L-17A, tumor necrosis factor (TNF)-α and interferon (IFN)-γ [23].

However, there are still some open issues that need to be considered when using PL for MSC clinical expansion. First of all there is still a lack of consensus on the standardization of method(s) used for hPL production, because it has been reported that the final concentration of cytokines and growth factors eventually affecting both functional and phenotypic proprieties of MSCs is strictly correlated to the preparation method [24]. In fact, several PL preparation methods are described in the literature. The differences start from the source material for PL preparation that is the platelet concentrate, which could be derived from an apheresis product or buffy coat or platelet-rich plasma. PL could be also prepared from expired platelet units. Source material could be fresh, expired, irradiated and/or pathogen inactivated [25]. The most frequent preparation methods include repeated freeze/thaw cycles, direct platelet activation by calcium chloride, sonification or solvent/detergent treatment. Difference in anticoagulant as the ancillary material may also have an impact on the final PL product [26]. Moreover, the lack of standardization may contribute to an inability to transfer technology or compare results of clinical trials performed by different groups.

Another issue that must be considered in the use of PL is the regulatory context, as the classification of PL as a blood component for nontransfusion use is still being discussed. As a matter of fact, the collection method, the product definition and the purpose of use are specified by EU GMP annex 14 and by the current transfusion regulations in different European countries but, in both cases, no specific indications for PL use as cell culture supplement are given [27, 28]. Consequently PL can still be considered as belonging to the group of ancillary materials for the manufacturing of cellular therapies [29].

Recently, the lack of consensus on the PL release criteria has also been discussed [30]. In fact, even if some studies [31] suggest a major role of specific cytokines/growth factors for MSC expansion, their final concentration is linked both to the production method and to the number of units pooled as the platelet starting concentration. Therefore, standardized release criteria should include the number of units pooled, platelet starting concentration [32], endotoxin content, absence of viral and bacterial contamination, and finally a concentration of specific growth factors for each PL batch produced. On this issue the necessity to identify a panel of specific molecules significantly affecting MSC main characteristics can be achieved, for example by a multiplex assay for simultaneous detection of cytokines, chemokines and growth factors [23].

However, there are some commercial alternatives to PL. Even if studies reported good results in increasing BM-MSC proliferation without compromising chromosomal stability, immunophenotype and immunomodulatory proprieties [33, 34], there are still some problems related to their use mostly due to costs, exact composition and GMP grade/clinical grade production.

As an Agenzia italiana del Farmaco (AIFA) authorized cell factory, we report our experience in using PL produced by Azienda Ospedaliero Universitaria (AOU) Meyer Transfusion Service for BM-MSC expansion according to a GMP grade protocol.

## Methods

### PL production

PL was produced by AOU Meyer Transfusion Service from whole blood-derived pooled platelet concentrates (PCs). Blood donors were tested according to guidelines for the preparation of blood and blood components and the use of blood products (Hemotherapy Guidelines) according to Decreto Ministeriale 2/11/2015 [28] of the Italian transfusion law. Whole blood from healthy volunteer donors was centrifuged and separated to obtain buffy coats (BCs). Five fresh BCs were pooled within 24 h of collection in 250 ml of additive solution (Composol PS®; Fresenius Kabi) and separated to obtain PC using a CompoStop®Flexible kit (Fresenius Kabi). Eight PCs were pooled together to obtain pooled PC (PPC) and the PPC was subjected to two centrifugation steps (one at 400 × *g* for 9 min at 22 °C, and one at 457 × *g* for 30 min at 4 °C; HERAEUS CRYOFUGE 16; Thermo Scientific). Supernatants were collected after centrifugation and one filtration step, in two 600-ml BioBags (Fresenius Kabi), and resuspended in an appropriate volume of thawed fresh-frozen AB group plasma to achieve a final platelet concentration between 1.5 × 10^6^ and 2.4 × 10^6^ platelets/μl. PPCs were stored overnight at −80 °C and subjected to three repeated freezing/thawing cycles and two centrifugation steps at 4579 × *g* for 10 min at 20 °C. Finally the supernatant, about 1000 ml of total volume, was divided into 25-ml aliquots and stored at −80 °C until use. PL is then subjected to another two filtration steps using 0.45-μm and 0.22-μm filters (Durapore® membrane Stericup and Steritop filtration systems; Millipore) when added to complete culture medium, and to a sterility test (BactALERT 3D system; Biomerieux) and endotoxin test (LAL portable PTS system; Charles River). PL batches were released for clinical-grade use only if negative for the following infectious disease markers: HIV 1 + 2 by chemiluminescent microparticle immunoassay (CMIA) and nucleic acid test (NAT), HCV by CMIA and NAT, hepatitis B surface antigen (HBsAg) by CMIA, HBV by NAT and *Treponema pallidum* by CMIA. Some examples of PL batches produced between 2015 and 2017 are presented in Table 1.

**Table 1:** Platelet lysate batches produced between 2015 and 2017

Batch ID	Platelet concentration (platelets/μl)	Batch final volume (ml)
I104215700001	1.9 × 10^6^	1050
I104215700079	2.26 × 10^6^	1034
I104215700078	2.27 × 10^6^	1000
I104215700053	2 × 10^6^	1032
I104215700039	1.89 × 10^6^	1076
I104216700061	1.6 × 10^6^	1002
I104217700035	1.8 × 10^6^	1045
I104217700010	1.6 × 10^6^	1012
I104217700061	1.71 × 10^6^	1015

*n* = 9. Mean platelet concentration = 1.89 × 10^6^ μl ± 0.25. Mean final volume = 1029.5 ± 24.9 ml

### Isolation and in-vitro expansion of BM-MSCs

BMNCs were harvested from the iliac crest of 12 donors (mean age 25) and equally split into two cellular culture conditions at a seeding density of 10,000 cells/cm^2^ [1]: Dulbecco’s modified Eagle medium high glucose (DMEM) (Invitrogen, Thermofisher) containing 10% of FBS (BioWest); and DMEM containing 5% PL and heparin 40 IU/ml (EPSOCLAR 5000 U.I./5 ml). After 5 days, nonadherent cells were removed and the adherent cells were refed every 3 days. When adherent cells reached confluence they were detached by adding TrypLE™ Select 10X (Thermofisher Scientific), counted with Bürker’s chamber and replated until passage 4 (P4) at 5000 cells/cm^2^. All experiments where performed at passage 3 or 4 (P3 or P4).

Experiments where performed at passage 3 or 4 because these correspond to the maximum number of expansions allowed by our clinical protocol and Investigational Medicinal Product Dossier (IMPD). Our IMPD is based on intravenous administration of third-party bone marrow-derived MSCs expanded in platelet lysate, in pediatric patients experiencing steroid-resistant grade II–IV graft-versus-host disease (GVHD). The clinical protocol is going to be authorized by the National Authorities for Phase I trials (Istituto Superiore di Sanità (ISS)). Moreover the protocol allows a minimum of two MSC infusions/patient and each MSC infusion aimed at reaching 1 ± 0.5 × 10^6^ cells/kg of recipient body weight. The clinical protocol has been developed for pediatric patients to provide a satisfactory cell dose within the fourth expansion passage (P4), avoiding long-term in-vitro culture effects [35].

### Evaluation of MSC proliferation

To evaluate cellular growth, the cell growth rate was expressed in terms of population doublings (PDs) calculated from P1 to P3 using the following formula:

Cumulative PD = (log(*n*) – log(*n*_0_)) / log2,

where *n* is the number of cells detached and *n*_0_ is the number of cells seeded.

### Evaluation of MSC immunophenotype

To evaluate the MSC immunophenotype a flow cytometer analysis was performed at passage 4. After the detachment, cells where washed with Dulbecco’s phosphate buffered saline (DPBS 1× without Ca^2+^/Mg^2+^; Euroclone) and briefly incubated with the following conjugated monoclonal antibodies and isotype controls for 15 min at room temperature: CD90 FITC, CD105 PE, CD73 PE, HLA-DR PE-Cy7, CD45 APC-Cy7, CD14 APC-Cy7, CD19 PE-Cy7,CD34 APC (Becton Dickinson), APC-Cy7 IGg_1_ isotype control, APC-Cy7 IGg_2_ isotype control, PE-Cy7 IGg_1_ isotype control, PE-Cy7 IGg_2_ isotype control, FITC IGg_1_ isotype control, PE IGg_1_ isotype control and APC IGg_1_ isotype control (BD Pharmingen). The vitality dye 7-aminoactinomycin D (7-AAD; BD Pharmingen) was also added to discriminate dead cells during flow cytometry analysis. After incubation, cells were washed, resuspended in DPBS and then analyzed by flow cytometry on a FACS Canto II (Becton Dickinson) equipped with the DIVA software program.

### Adipogenic differentiation

MSCs at passage 4 were utilized for adipogenic differentiation assay. Briefly, MSCs derived from FBS and PL cultures were washed with DPBS and plated in a 24-multiwell flat-bottom plate (Euro Clone) at a seeding concentration of 3 × 10^3^ MSCs/cm^2^, with the StemPro™ Adipogenesis Differentiation Kit (Gibco™, ThermoFisher) following the manufacturer’s instructions. The medium was changed every 3 days and plates were observed under the microscope for the development of lipid droplets. Adipogenic differentiation was confirmed by Oil Red staining (Oil Red O; Sigma-Aldrich) after 15 days of culture in differentiation medium.

### Osteogenic differentiation

MSCs at passage 4 were utilized for osteogenic differentiation assay. Briefly, MSCs derived from FBS and PL cultures were washed with DPBS and plated in a 24-multiwell flat-bottom plate at a seeding concentration of 3 × 10^3^ MSCs/cm^2^, with the StemPro™ Osteogenesis Differentiation Kit (Gibco™, ThermoFisher) following the manufacturer’s instructions. The medium was changed every 3 days and osteogenic differentiation was confirmed by Alizarin Red staining of the calcium matrix (Alizarin Red S; Sigma-Aldrich) after 21 days of culture in differentiation medium.

### Chondrogenic differentiation

MSCs at passage 4 were utilized for chondrogenic differentiation assay. MSCs derived from FBS and PL cultures were washed with DPBS and plated in a 24-multiwell flat-bottom plate at a seeding concentration of 16 × 10^3^ MSCs/well, with the StemPro™ Chondrogenesis Differentiation Kit (Gibco™, ThermoFisher) following the manufacturer’s instructions. The medium was changed every 3 days and chondrogenic differentiation was confirmed by Alcian Blue staining of glycosaminoglycans (Alcian Blue 8GX; Sigma-Aldrich) after 15 days of culture in differentiation medium.

### Mixed leukocyte reaction

In order to assess the immunomodulatory potential of MSCs in both culture conditions, a mixed leukocyte reaction (MLR) was performed. Peripheral blood mononuclear cells (PBMCs) from five different donors were obtained by Ficoll (Lymphosep, Lymphocyte Separation Media; BioWest) gradient centrifugation and resuspended in RPMI1640 medium (Euroclone) supplemented with 10% FBS.

Stimulator mononuclear cells (S) were irradiated at 60 Gy before being cultured with responder PBMCs (R). Then 200 × 10^3^ responders and 100 × 10^3^ stimulators were mixed with different concentrations of MSCs to obtain different concentration ratios (1:5, 1:10, 1:20, 1:100) in flat-bottomed 96-well plates (Corning) for 6 days. As controls, a polyclonal stimulation with purified anti-CD3/28 antibodies (BD Pharmingen) was also performed on PBMC responders in the presence of MSCs. Proliferation was measured on day 6 by carboxyfluorescein diacetate succinimidyl ester (CFDA-SE) (Vybrant CFDA SE Cell Tracer Kit; Thermofisher) labeling of responder PBMCs. A FACS analysis on flow cytometry was performed on responder PBMCs after incubation with the following conjugated monoclonal antibodies: CD25 PE, CD45 APCCy7, CD3 APC and HLA-DR PECy7 (Becton Dickinson). The immunomodulatory effect is evaluated as a percentage of proliferation (% CFDA-SE-positive cells) in the presence/absence of MSCs on 7-AAD^−^ CD45/CD3^+^ cells.

### T-regulatory cell induction

To assess the capacity of MSCs to induce the generation of T-regulatory (Treg) cells, PBMC-MSC cocultures have been set up. In short, 500 × 10^3^ PBMCs from five different donors obtained by Ficoll gradient centrifugation were plated in the presence of 50,000 allogeneic MSCs in RPMI 10% FBS with IL-2300 U/ml in a 24-multiwell flat-bottomed plate for 1 week of culture [36]. At day 7, PBMCs were harvested and analyzed by flow cytometry after incubation with the following conjugated monoclonal antibodies: CD25 FITC, CD127 PE, CD4 APC, CD3 PECy7 and CD45 APCCy7 (Becton Dickinson). Treg cell induction by MSCs was evaluated as the percentage of CD25^High^/CD4^+^/CD127^Low/−^ PBMCs [37].

### Evaluation of relative telomere length

In order to assess senescence possibly induced by PL on MSCs [38], evaluation of the relative telomere length (RTL) was performed. The study of RTL was conducted on MSCs derived from both culture conditions by a flow-FISH assay that combines fluorescent-hybridization in situ and flow cytometry with a TELOMERE-PNA kit/FITC (Dako Cytomation) using a FITC-conjugated peptide nucleic acid (PNA) probe. A human erythromyeloblastoid leukemia cell line (K562, provided by Laboratory of Cellular Manipulation, Hospital of Modena and Reggio Emilia, Italy) with known telomere length and DNA index were used as control. MSCs were equally mixed with K562 and subjected to DNA denaturation, hybridization and DNA staining with propidium iodide according to the manufacturer’s instructions. Cells were analyzed by flow cytometry on a FACS Canto II (Becton Dickinson). Samples hybridized with Telomere PNA Probe/FITC exhibit a fluorescence signal in FL1 which is higher than the background/autofluorescence signal obtained from the control of the same cells hybridized with the hybridization solution without probe. Cells gating for flow cytometry included cells in G_0_–G_1_ phases and gated cells were displayed for analysis in the FL3-height versus FL1-height dot plot. Results are reported as the RTL value calculated as the ratio between the telomere signal of each sample and control (ctrl) cells (K562 line) with correction for the DNA index of G_0_–G_1_ cells by the following formula: $\mathrm{RTL}=\frac{\left(\mathrm{meanFL}{1}_{\exp .\mathrm{cells}}\mathrm{withPNA}\right)\hbox{-} \left(\mathrm{meanFL}{1}_{\exp .\mathrm{cells}}\mathrm{withoutPNA}\right)}{\left(\mathrm{meanFL}{1}_{\mathrm{ctrlcells}}\mathrm{withPNA}\right)\hbox{-} \left(\mathrm{meanFL}{1}_{\mathrm{ctrlcells}}\mathrm{withoutPNA}\right)}\times 100$.

### Statistical analysis

Statistical tests were performed using StatPlus pro 2009 software (AnalystSoft, Walnut, CA, USA). Data are presented as the mean ± SD. Statistical differences were calculated using a *t* test for paired data. Differences were considered significant at *p* < 0.05 for each test.

## Results

### PL increases MSC proliferation and induces morphological changes

**Fig. 1:**
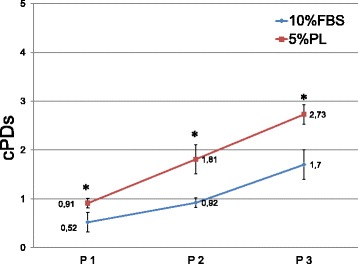
MSC cumulative population doublings calculated from P1 to P3 in culture conditions DMEM 10% FBS versus DMEM 5% PL. *n* = 12. Data presented as mean ± SD. *p < 0.05. cPD cumulative population doubling, FBS fetal bovine serum, PL platelet lysate

As reported previously by other authors, PL increases MSC proliferation evaluated in terms of population doublings. As illustrated in Fig. 1, PL significantly increases MSC population doublings at each passage (from P1 to P3) compared to MSCs cultured in DMEM 10% FBS. Even if evaluated only at passage 3, it can be stated that PL increases MSC proliferation starting from an early passage from isolation (P1). Moreover, as discussed by others [39, 40], there are significant differences in MSC proliferation with PL as it facilitates expansion for more than 20 population doublings and more than 10 passages (P10).

**Fig. 2:**
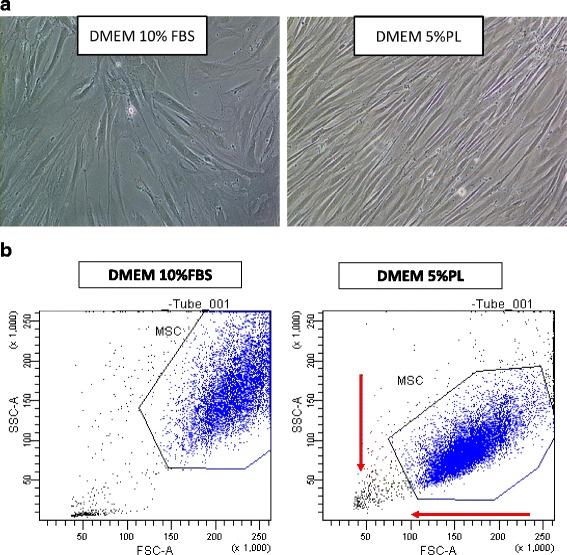
Morphological changes induced by PL (representative case). **a** When observed by inverted microscope (10×), MCSs cultivated in presence of PL appear smaller, more refractive and less attached to plastic support than MCSs cultured with FBS. **b** Morphological changes confirmed by flow cytometry analysis of FSC and SSC, as cells cultured in PL showed reduction of FSC and SSC rate representative of less complexity and smaller dimensions. DMEM Dulbecco’s modified Eagle medium, FBS fetal bovine serum, FSC forward scatter, MSC mesenchymal stromal/stem cell, PL platelet lysate, SSC side scatter

At the same time, cells cultured in PL show a different morphology: in both culture conditions, cells exhibited the classical fibroblast-like morphology typical of MSCs; but in PL-supplemented media, cells were even more spindle shaped, elongated and showed denser cell bodies than MSCs from FBS-supplemented cultures. These morphological changes were also confirmed by flow cytometry analysis of forward scatter (FSC) and side scatter (SSC), as cells cultured in PL showed a reduction of FSC and SSC rates representative of less complexity and smaller dimensions (Fig. 2a, b).

### PL does not affect MSC immunophenotype

As illustrated in Table 2, PL does not affect the MSC immunophenotype as cells expanded in two different media (DMEM 10% FBS and DMEM 5% PL) showed the normal MSC phenotype. In fact, in both culture conditions, after FACS analysis at passage 3, MSCs meet the ISCT criteria as they are negative (< 2%) for CD14, CD19, CD45 and CD34 and positive for CD73, CD90 and CD105 (> 90%) without significant differences between the two media.

**Table 2:** MSC immunophenotypic analysis of PL-MSCs versus FBS-MSCs

	CD90	CD105	CD73	HLA-DR	CD45	CD14	CD19	CD34
FBS-MSCs	95.5 ± 1.6	96.8 ± 1.5	98.7 ± 1.1	2.9 ± 0.8	0.3 ± 0.1	1.3 ± 0.5	1.6 ± 0.5	0.6 ± 0.1
PL-MSCs	96.6 ± 0.6	93.2 ± 2.6	97.7 ± 1.5	1.5 ± 0.3	0.2 ± 0.1	1.2 ± 0.2	1.4 ± 0.1	0.5 ± 0.1

Values expressed as mean percentage ± SD of positive cells for MSC antigen expression

*n* = 12. Cells analyzed at the third passage for each experimental condition. No significant differences were observed (*p* > 0.05)

*FBS* fetal bovine serum, *MSC* mesenchymal stromal/stem cell, *PL* platelet lysate

### PL does not affect MSC differentiation potential

**Fig. 3:**
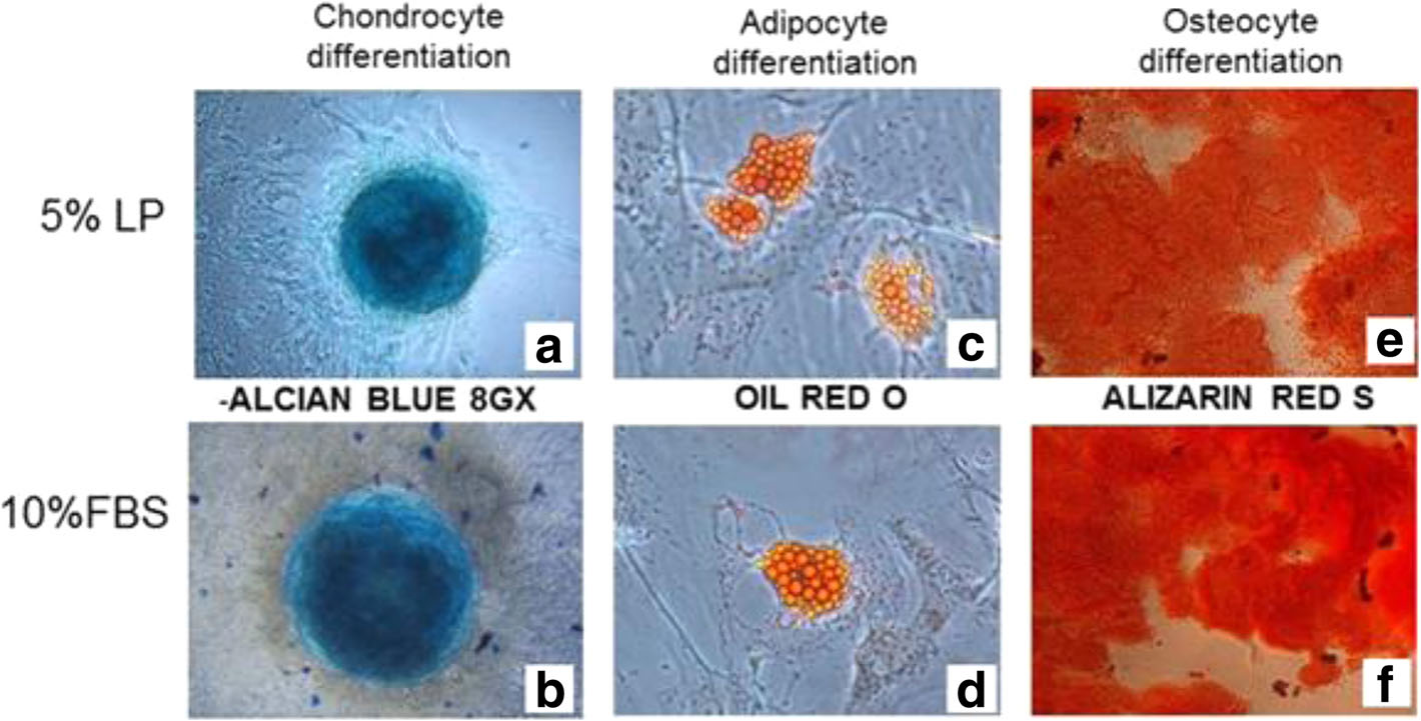
MSC differentiation potential assays. MSC differentiation potential assay after 15 or 21 days of specific induction in both culture conditions. **a, b** Alcian Blue staining shows hyaluronic acid for chondrocytes, **c, d** Oil Red O shows intracytoplasmatic vacuoles in adipocytes and **e, f** Alizarin Red S staining shows presence of calcium matrix in osteoblasts, respectively, in PL-MSCs and FBS-MSCs. *n* = 12. FBS fetal bovine serum, LP platelet lysate

MSCs cultured in the two different conditions showed multipotent capacity as all samples at P4 differentiated into osteoblasts, adipocytes and chondrocytes, as shown in Fig. 3. After 21 days of osteogenic differentiation, mineralization was observed in all cultures, as seen by the Alizarin Red S staining. Cultures supplemented with adipogenic stimulus for 15 days underwent morphological changes from a fibroblast-like appearance to round cells with distinct lipid vacuoles in the cytoplasm, which stained positive with Oil Red O stain. Chondrogenic differentiation could be observed in both conditions after 15 days of culture with chondrogenic stimulus as micromass development stained with Alcian Blue.

### PL does not affect MSC immunomodulatory potential

**Fig. 4:**
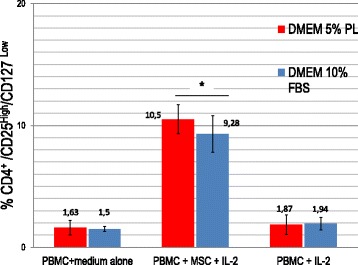
Induction of T-regulatory cell population by MSCs cultured in PL or FBS-containing medium. Data presented as mean ± SD with *n* = 10. T_reg_ cell induction by MSCs evaluated as percentage of CD25^High^/CD4^+^/CD127^Low/−^ PBMCs after 7 days of coculture. No significant differences found between MSCs cultured in PL or FBS-containing medium. In both culture conditions at day 7, percentage of CD4^+^/CD25^high^/CD127^Low/−^ T cells was significantly higher (*p < 0.05) in cocultures with MSCs compared to PBMCs alone. DMEM Dulbecco’s modified Eagle medium, FBS fetal bovine serum, IL interleukin, MSC mesenchymal stromal/stem cell, PBMC peripheral blood mononuclear cell, PL platelet lysate

**Fig. 5:**
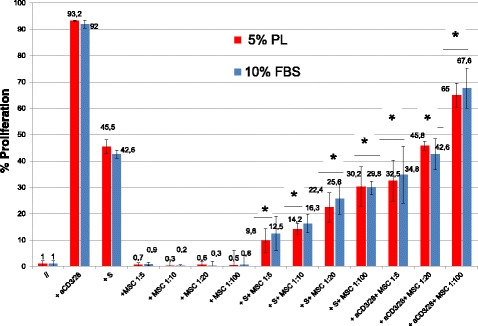
Inhibition of MLR by MSCs cultured in PL or FBS-containing medium. Data presented as mean ± SD with *n* = 10. Inhibition of PMBC responder (R) proliferation in presence of both allogeneic (PBMC stimulator (S)) and polyclonal (aCD3/28) stimuli evaluated as percentage of proliferation (% CFDA-SE-positive cells) in presence/absence of MSCs on 7-AAD^−^ CD45/CD3^+^ cells. No significant differences found between MSCs cultured in PL or FBS-containing medium. In both culture conditions, MSCs significantly reduce PBMC proliferation compared to PBMCs alone (*p < 0.05) in a dose-dependent manner. FBS fetal bovine serum, MSC mesenchymal stromal/stem cell, PL platelet lysate

As reported in Figs. 4 and 5, MSCs cultured in PL retain the ability to induce a Treg cell population and are able to inhibit PBMC proliferation in a mixed leukocyte reaction (MLR), in the presence of both allogeneic and polyclonal stimuli. The immunomodulatory functions of MSCs were evaluated by MLR assays after cell expansion in both culture conditions (DMEM 10% FBS and DMEM 5% PL). The proliferation of allogeneic (presence of third-party PBMC stimulator) and polyclonal (anti-CD3/28 antibodies) stimulated PBMCs cocultured with MSCs was compared to PBMC proliferation in the absence of MSCs. Coculture of MSCs with PBMCs significantly reduced the proliferation of PBMCs as compared to PBMCs alone (*p* < 0.05). The use of PL or FBS as culture supplements during cell expansion did not affect the ability of MSCs to reduce PBMC proliferation evaluated by the decrease of CFDA-SE fluorescence. Moreover, it was possible to observe how PL-MSCs retain the ability to inhibit PBMC proliferation in a dose-dependent manner when compared to FBS-MSCs.

The induction of Treg cells was studied in cocultures of PBMCs and MSCs expanded in two different culture conditions (DMEM 10% FBS and DMEM 5% PL). At day 7 the percentage of CD4^+^/CD25^high^/CD127^Low/−^ T cells was significantly higher (*p* < 0.05) in the population that was cultured with MSCs compared to PBMCs that were cultured without MSCs. As reported in Fig. 4, it is possible to observe how PL-MSCs and FBS-MSCs show the same ability to induce a Treg population without significant differences (*p* > 0.05). Treg induction was also evaluated by Foxp3 assay (Additional file 1: Figure S1).

### PL does not affect MSC relative telomere length

**Fig. 6:**
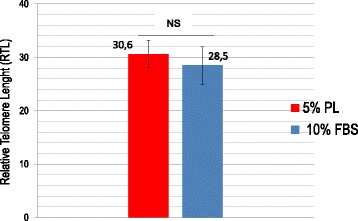
MSC relative telomere length at passage 4. Data presented as mean ± SD with *n* = 12. RTL value calculated as ratio between telomere signal of each sample and control cells (K562 line) with correction for DNA index of G_0_–G_1_ cells. No significant differences observed (*p* > 0.05). FBS fetal bovine serum, NS not significant, PL platelet lysate

The relative telomere length (RTL) remained unaffected by PL. No significant differences were found at passage 4 (*p* > 0.05) in both culture conditions, as reported in Fig. 6. Even if data are related to early culture passages (P4) which coincide with a short period of culture (at most 40 days of culture), it is possible to observe that even if PL significantly increases cell proliferation, at the same time it does not determine significant alterations in telomere length when compared to MSCs expanded with FBS.

## Discussion

Mesenchymal stem/stromal cells (MSCs) represent one of the most promising tools in cellular therapy and regeneration. The increasing use of MSCs as ATMP has led to production processes that need to meet GMP and that need to achieve a therapeutic target through ex-vivo expansion. Fetal bovine serum (FBS) has long been the gold standard medium supplement for laboratory-scale MSC culture but, due to a poorly characterized composition and related zoonotic risk of infection, the regulatory authorities discourage the use of FBS for GMP grade MSC expansion. In recent years platelet lysate (PL), a source of bioactive molecules and growth factors, has been investigated and proposed as a substitute for FBS. Despite the large number of publications, the use of PL for ex-vivo expansion of MSCs is still considered a “hot topic” for those who work with MSCs as an advanced cell therapy product according to a GMP grade protocol. The main issues related to this use are principally the lack of consensus on the standardization of the method(s) for PL production, the regulatory context for the classification of PL as a blood component for nontransfusion use is still being discussed, and finally the necessity to achieve a consensus for PL release criteria. In this context, the research and development phase represents an essential step to standardize PL production: for example, some proposals might include a multiplex assay for simultaneous detection and quantification of cytokines, chemokines and growth factors in each PL batch produced. An in-vitro test on MSCs versus MSCs cultured with FBS still represents the best way to test PL batches. In this study we report our experience as a cell factory using PL produced by our internal transfusion service for MSC expansion. As suggested by other authors, we focused on MSC surface marker expression, MSC immunomodulatory potential, differentiation potential and relative telomere length.

Results obtained confirm the literature data as PL increases cell proliferation without affecting MSC immunophenotype, immunomodulatory potential, differentiation potential and relative telomere length. The PL production method developed by our supplier, the AOU Meyer Transfusion Service, is standardized as they can produce batches with similar volume (about 1000 ml) and starting platelet concentration. Even if some studies are in progress in order to characterize PL composition, this aspect allows the cell factory to expand a single MSC batch (about 250 × 10^6^ MSCs/batch) with a single PL batch, minimizing the biological variability of the product itself and the possible impact on the MSC batch. We have not yet experienced a PL batch that did not work for MSC expansion as we collaborate with our supplier to reach a standardized PL production, especially focusing on thrombocyte concentration. As reported by several studies [41, 42], the concentration of thrombocytes, which is directly related to growth factor concentration, is crucial for MSC manufacturing. For example, Lange et al. [42] evaluated different thrombocyte concentrations as a 5% supplement in basal medium to evaluate the effect on MSC proliferation. The study concluded that a platelet concentration below 1.5 × 10^6^ platelets/μl significantly reduced the pro-proliferative effect. The mean platelet concentration of our PL batches used for MSC clinical expansion under GMP conditions is 1.89 × 10^6^ μl. Moreover, according to the PL batch quality certificate as a release criterion, platelet concentrations must be between 1.6 × 10^6^ and 2.4 × 10^6^ platelets/μl for each PL batch to ensure a satisfactory MSC expansion. As further supporting data we also found a moderate correlation between the number of platelets in each PL batch and the MSC population doubling analyzed from P1 to P3. MSC cumulative population doublings (cPDs) at passages 1, 2 and 3 have been related to the platelet concentration of each PL batch by Spearman’s rank correlation coefficient (*r*_s_) and we found a moderate correlation (*r*_s_ at passage 1 = 0.7564, *r*_s_ at passage 2 = 0.6543, *r*_s_ at passage 3 = 0.6764; data not shown).

## Conclusions

Platelet lysate can be considered a safe alternative to FBS, providing a human-based, xeno-free culture system for in-vitro mesenchymal stem cell expansion according to a GMP grade protocol. Our experience confirms the literature data: a large number of MSCs for clinical applications can be obtained by expansion with PL, without affecting the MSC main features. Our experience underlines the benefits of a close collaboration between the PL producers (transfusion service) and the end users (the cell factory) in a synergy of skills and experiences that, although coming from two “different worlds”, end up finding a meeting point in the research and development of a standardized final product. The culture medium is only one building block inside the complexity of the GMP-compliant MSC manufacturing process: despite the intensified research work in the translational field, only agreement on standardized protocols can lead to scaling up this process to produce billions of cells needed for clinical trials. Some studies have demonstrated the possibility to make a process transfer of mesenchymal stromal cell production from monolayer to microcarrier culture using human PL [43]. We believe that there is still the necessity to define the regulatory context, and the necessity to achieve a consensus for PL production method and PL release criteria. Once consensus has been reached on these aspects, it could be interesting collecting the transfusion centers’ experience (as PL producer) and the cell factories’ experience (as end users) and with the definition given by competent authorities carry out a technology transfer to pharmaceutical industries. In this way, standardized and large-scale production could be developed and PL could be assimilated to other industrial blood derivatives.

## Additional file

Additional file 1: **Figure S1.** Evaluation of Treg cell induction by MSCs expanded in PL with two different methods. Data presented as mean ± SD with *n* = 6. Treg induction evaluated after 7 days of coculture with MSC as % CD4^+^/CD25^High^/CD127^Low^ PBMCs versus %CD4^+^/CD25^High^/Foxp3^+^ PBMCs. No significant differences (*p* > 0.05) found between the two methods concerning identification of Treg cells. Foxp3 staining of PBMCs performed with the BD Pharmingen™ Anti-Human FoxP3 Staining Kit. MSCs cultured with PL were able to induce the Treg cell population if compared with PBMCs alone (*p < 0.05). (PDF 179 kb)

## Abbreviations

ATMP: Advanced therapy medicinal products
BM: Bone marrow
FBS: Fetal bovine serum
GMP: Good manufacturing practice
MSC: Mesenchymal stromal/stem cell
PBMC: Peripheral blood mononuclear cell
PL: Platelet lysate
