# Supplementary figures and images for: Human platelet lysate in mesenchymal stromal cell expansion according to a GMP grade protocol: a cell factory experience
Source: Stem Cell Res Ther. 2018 May 2;9:124. doi: 10.1186/s13287-018-0863-8 (PMC5930506; doi:10.1186/s13287-018-0863-8)

% of cells expressing specific markers

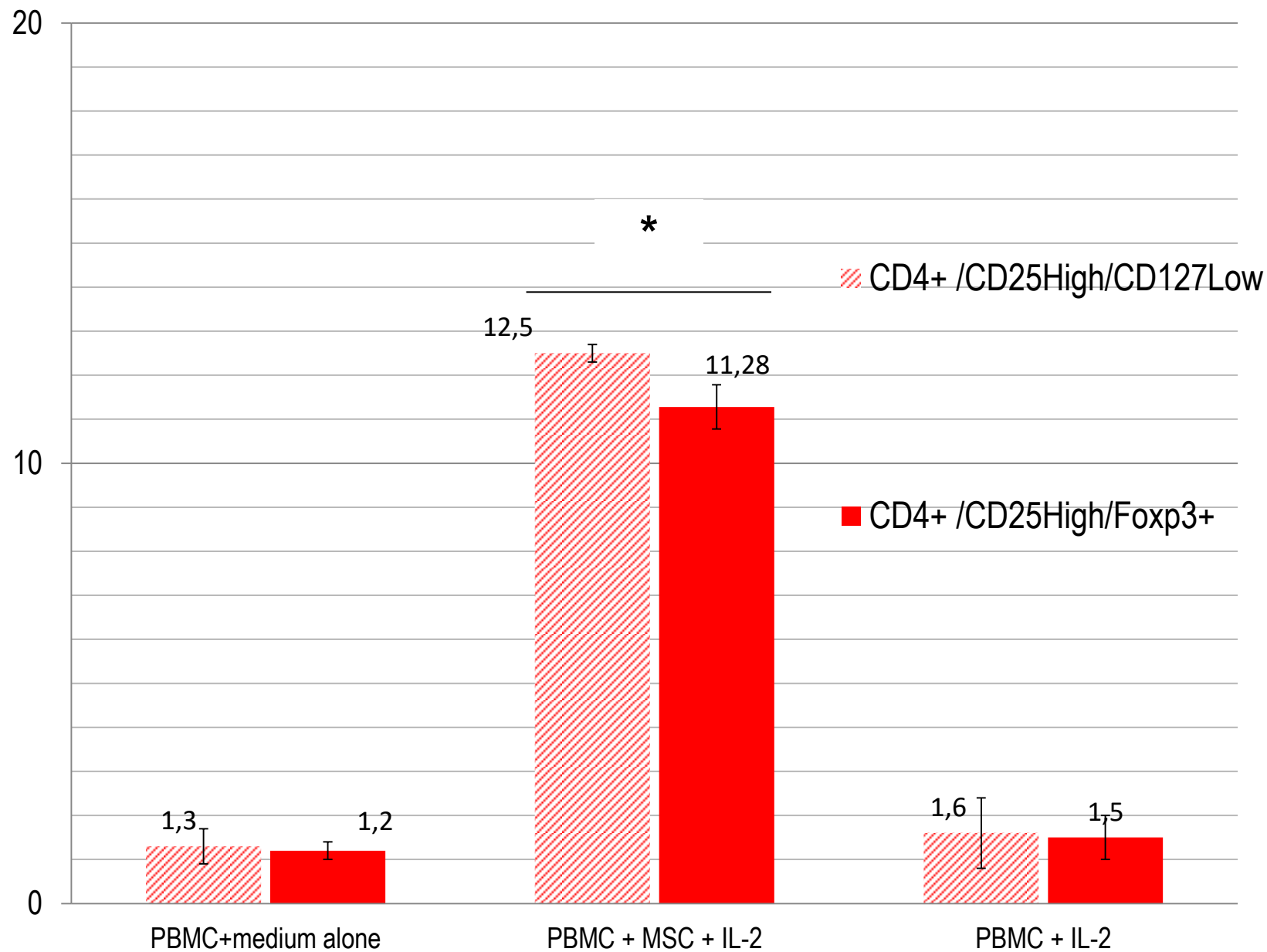

Supplement: Supplementary file 1 — Figure S1. Evaluation of Treg cell induction by MSCs expanded in PL with two different methods. Data presented as mean ± SD with n = 6. Treg induction evaluated after 7 days of coculture with MSC as % CD4+/CD25High/CD127Low PBMCs versus %CD4+/CD25High/Foxp3+ PBMCs. No significant differences (p > 0.05) found between the two methods concerning identification of Treg cells. Foxp3 staining of PBMCs performed with the BD Pharmingen™ Anti-Human FoxP3 Staining Kit. MSCs cultured with PL were able to induce the Treg cell population if compared with PBMCs alone (*p < 0.05). (PDF 179 kb) [file 13287_2018_863_MOESM1_ESM.pdf]
